# The immunopathogenesis of SARS‐CoV‐2 infection in children: diagnostics, treatment and prevention

**DOI:** 10.1002/cti2.1405

**Published:** 2022-07-25

**Authors:** Harsita Patel, Andrew McArdle, Eleanor Seaby, Michael Levin, Elizabeth Whittaker

**Affiliations:** ^1^ Department of Infectious Disease, Section of Paediatric Infectious Disease Imperial College London London UK; ^2^ Genomic Informatics Group University of Southampton Southampton UK; ^3^ Translational Genomics Group Broad Institute of MIT and Harvard Cambridge Massachusetts USA; ^4^ Department of Paediatrics Imperial College Healthcare NHS Trust London UK

**Keywords:** antibody, COVID‐19, immunology, MIS‐C, paediatrics, SARS‐CoV‐2

## Abstract

Symptoms and outcomes for paediatric COVID‐19 differ vastly from those for adults, with much lower morbidity and mortality. Immunopathogenesis drives severe outcomes in adults, and it is likely that age‐related differences in both the innate and specific immune responses underlie much of the variation. Understanding the protective features of the paediatric immune system may be crucial to better elucidate disease severity in adult COVID‐19 and may pave the way for novel therapeutic approaches. However, as well as uncommon cases of severe paediatric acute COVID‐19, there have been children who have presented with delayed multisystem inflammation, including cardiac, gastrointestinal, skin, mucosa and central nervous system involvement. The occurrence of coronary artery aneurysms has drawn comparisons with Kawasaki Disease, but similarities with the inflammatory phase of adult acute COVID‐19 have also been drawn. In this review, we summarise findings from studies investigating pre‐existing immunity, cytokine profiles, innate, B‐cell, antibody, T‐cell and vaccine responses in children with acute COVID‐19 and multisystem inflammation, compared with COVID‐19 adults and controls. We further consider the relevance to therapeutics in the context of limited evidence in children and highlight key questions to be answered about the immune response of children to SARS‐CoV‐2.

## Graphical abstract

Children have experienced a very different disease profile with SARS‐CoV‐2 infection compared with adults. Understanding differences in the immune responses with age will contribute to our understanding of the prevention, diagnosis and treatment of COVID‐19 disease in all age groups.

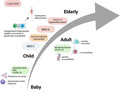

## Introduction

Children have experienced different disease phenotypes with SARS‐CoV‐2 compared to adults, with the vast majority experiencing only asymptomatic or mild disease, with a broad range of symptoms, and a subset developing a postinfectious multisystem inflammatory syndrome, rarely seen in adults.

It has been clear from the start of the pandemic that an understanding of the immunopathogenesis of COVID‐19 would be essential to control it—through rapid diagnostics, effective immunomodulatory treatment and, most crucially, vaccine prevention. Understanding the protective immunology that has led to such a mild disease course in children, and the factors that are driving rare inflammatory reactions, is vital for early, effective and safe management. Few studies have carried out a detailed comparative analysis of the immune response in children and adults with SARS‐CoV‐2‐related illness. Therefore, the fundamental questions of why most children suffer mild disease compared with the severe illness seen in a minority of children and how the immune system differs between mild and severe illness are unknown—ultimately leaving us with more questions than answers. In this review, we summarise the published literature to date and highlight the important questions that remain unanswered (Box 1).

Box 1 **Key questions that remain unanswered**

‐ What are the protective mechanisms resulting in mostly mild or asymptomatic COVID‐19 in children?

‐ Does the duration of immunity differ from infection versus vaccination?

‐ Is it better for children to develop ‘natural immunity’ given the low rate of severe disease?

‐ What are the genetic factors contributing to disease susceptibility in MIS‐C?

‐ How do genetic factors and other risk factors drive the immune dysregulation of MIS‐C?

‐ What is the role of endothelial health?

‐ Why are only children and young adults affected by MIS‐C?

‐ Is the inflammatory process in MIS‐C and the inflammatory phase of adult COVID due to similar mechanisms?

‐ How are the immunological disturbances of KD and MIS‐C related?

### Paediatric COVID: exposure and spectrum of disease

Children and young people (CYP) account for 1–2% of reported cases of SARS‐CoV‐2 infection worldwide.^1^ In population‐based screening using polymerase chain reaction (PCR) detection of SARS‐CoV‐2, the incidence is lowest in children under 10 years of age.^2^ Accordingly, SARS‐CoV‐2 seroprevalence increases with age.^3^ Children and young people appear to have lower susceptibility to infection with SARS‐CoV‐2, with one meta‐analysis reporting an odds ratio of 0.56 (95% Confidence Interval (CI), 0.37–0.85) for being an infected contact compared with adults.^4^

In addition, CYP experience less severe disease and the majority are asymptomatic or have mild disease; in one meta‐analysis including hospitalised and nonhospitalised children, the proportion of asymptomatic CYP ranged from 14.6% to 42%.^4^ Fever (46–64.2%) and cough (32–56%) are the most common reported symptoms, with other symptoms (rhinorrhoea, headache, fatigue, diarrhoea and vomiting) occurring less frequently (10–20%).^5^

One large multicentre observational study in the UK reported on 651 children admitted to hospital, with a median age of 4.6 years (Interquartile range (IQR) 0.3–13.7) of which 35% were under 1 year).^6^ Three phenotypes were identified: discrete respiratory illness, a mucocutaneous‐enteric illness and a less common isolated neurologic presentation. Paediatric intensive care (PICU) admission was required for 18% of patients. Although the severe disease is uncommon, risk factors for increased severity and mortality include age < 3 months, preterm birth and comorbidities, including neurodisability, underlying respiratory conditions and gastrointestinal conditions (GI).^6^ In the UK cohort, all children dying (*n* = 6) had co‐morbid conditions. Larger multinational surveillance studies also identified that age under 1 year and underlying medical conditions (including being ‘medically complex’ (40%), immunosuppressed (23%), obese (15%) and diabetic (8%) were associated with critical care admission and death.^7^

In April 2020, reports of an inflammatory condition with features overlapping with Kawasaki Disease and Toxic Shock Syndrome emerged in Italy and the UK. Subsequently, countries in Europe, the Americas and Asia have reported cases of this rare syndrome, now called Multisystem Inflammatory Syndrome in Children (MIS‐C) or Paediatric Inflammatory Multisystem Syndrome (PIMS), that is temporally associated with SARS‐CoV‐2 infection. Case definitions use criteria including clinical manifestations (fever, inflammation, organ dysfunction), elevated biochemical markers of inflammation and evidence of contact or infection with SARS‐CoV‐2, with the exclusion of another microbial cause.

**Figure 1:**
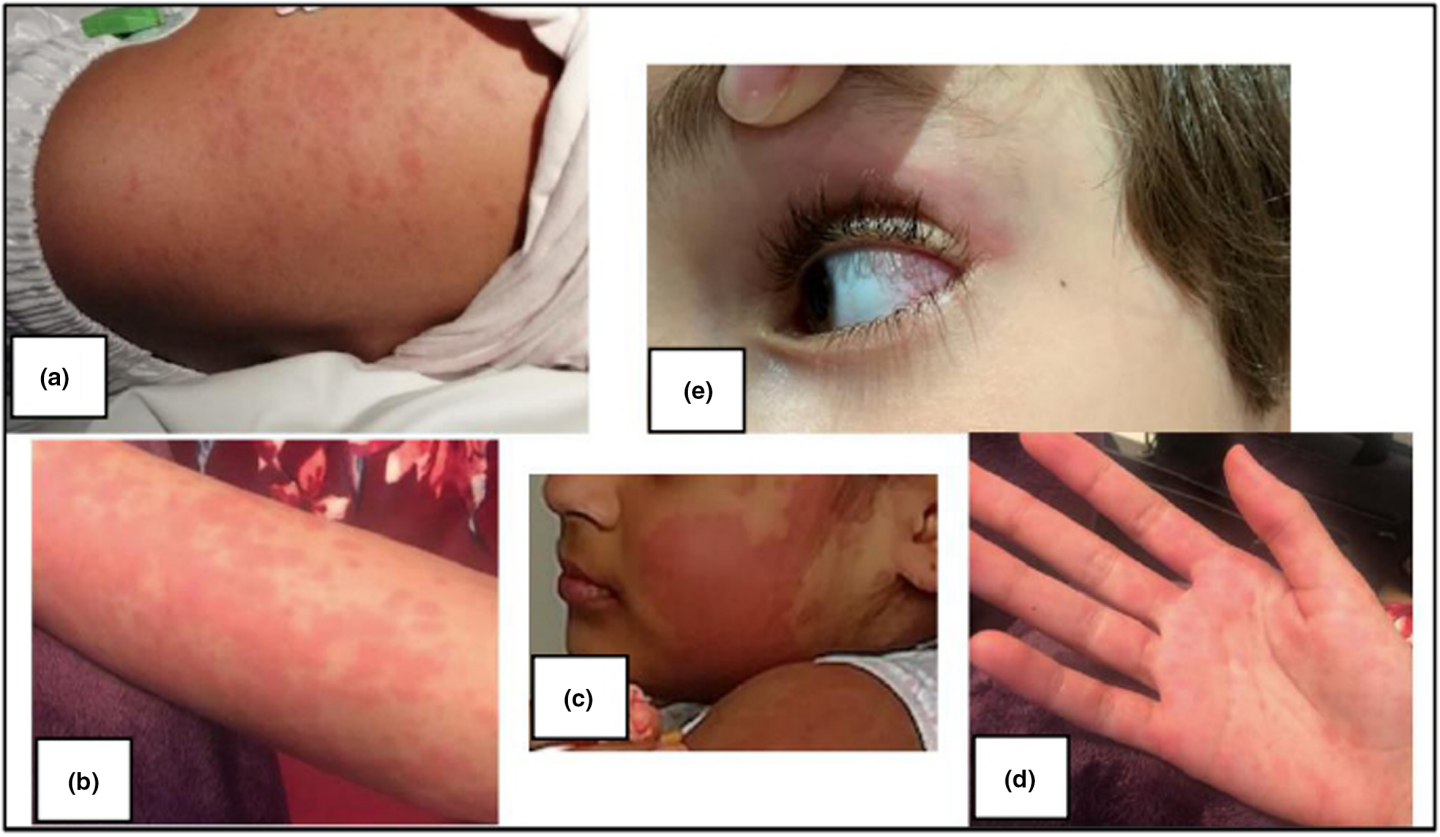
Clinical photographs (taken and published with consent) showing mucocutaneous features of multisystem inflammatory syndrome in children. **(a–d)** An erythematous maculopapular rash affecting the torso, arm, face and palms. **(e)** Nonpurulent conjunctivitis.

Children with MIS‐C present with fevers and a variety of mucocutaneous features (conjunctival injection, mucosal erythema, maculopapular rashes) (Figure 1), gastrointestinal involvement (abdominal pain, diarrhoea, vomiting), neurological involvement (confusion, headaches and lethargy) and concerningly, in 62% of cases, cardiac involvement.^8^ Characteristic laboratory findings include raised neutrophils and neutrophil‐to‐lymphocyte ratio, CRP, ferritin and D‐dimer, with low levels of haemoglobin, platelets, lymphocytes and serum albumin. Cardiac markers, including troponin and brain natriuretic peptide (BNP), are often elevated.^8^, ^9^, ^10^

From early data, the estimated incidence of MIS‐C in those younger than 21 years in the US is 2.1 per 100 000; however, this estimate is imprecise as the full disease spectrum is currently being determined and the true denominator is unknown.^11^In the largest reported cohort, the median age was 8.9 years (IQR 4.7–13.2); 58% were male and 1.9% died,^9^ compared with 1.4% for children with severe SARS‐CoV‐2 pneumonia.^9^, ^10^ As described in adults,^12^ children of ethnic minority groups appear over‐represented in cases of both SARS‐CoV‐2 infection and the multisystem inflammatory syndrome.^9^ It is intriguing that the incidence of MIS‐C is highest in children from Black African (34.8%), Caucasian (27.6%) and Hispanic origins (19.3%), and low in Asian ethnicities (8.1%).^13^ Contrastingly, KD predominantly affects children from East Asian ethnicities (330 per 100 000 in children of Japanese ancestry^14^).

Significant comorbidities are generally uncommon in this patient cohort and intriguingly children with primary immunodeficiencies^15^, ^16^ or on immunosuppressive treatment do not appear to experience more severe disease overall.^17^ However, obesity has been identified as a risk factor in the United States (US) cohort (36%).^9^ Across the UK, children with MIS‐C were five times more likely to be admitted to critical care than children with acute SARS‐CoV‐2, the majority (78%) with no comorbidities.^6^, ^18^ SARS‐CoV‐2 seropositivity is common (76%).

Gastrointestinal symptoms are reported in 80–100% of patients with MIS‐C.^8^ In the UK and US cohorts, cardiovascular complications also have been frequently reported (50 and 66.7%, respectively), with 14% of cases developing coronary artery aneurysms (CAA).^8^, ^9^ However, the majority of cardiovascular dysfunction and CAA (91 and 79%, respectively) normalised within 30 days,^9^ showing better resolution than KD.^19^

Of nearly 1700 patients with MIS‐C in one US cohort, 365 (22%) had documented neurologic involvement, 12% of whom developed life‐threatening conditions including severe encephalopathy, stroke or central nervous system (CNS) demyelination, and 11 (3%) died. Severe neurological involvement is associated with higher neutrophil‐to‐lymphocyte ratios,^20^ CRP, fibrinogen and D‐dimers^21^ than those without neurological involvement.

Although a number of studies exploring the pathophysiology of this disease are underway,^22^, ^23^ the mechanisms underlying MIS‐C remain poorly understood. Its relationship to KD, another paediatric acute inflammatory condition that causes CAA and sometimes shock, has been widely noted. Its occurrence several weeks after SARS‐CoV‐2 infection or exposure, suggests that MIS‐C may be mediated by an aberrant immune response, either through antibodies or T cells. Its rare occurrence and ancestral disparities suggest a genetic predisposition. There are many similarities between MIS‐C and the inflammatory phase of acute COVID‐19 infection in adults, leading to speculation that they have a similar underlying mechanism.

### Immunology overview

**Figure 2:**
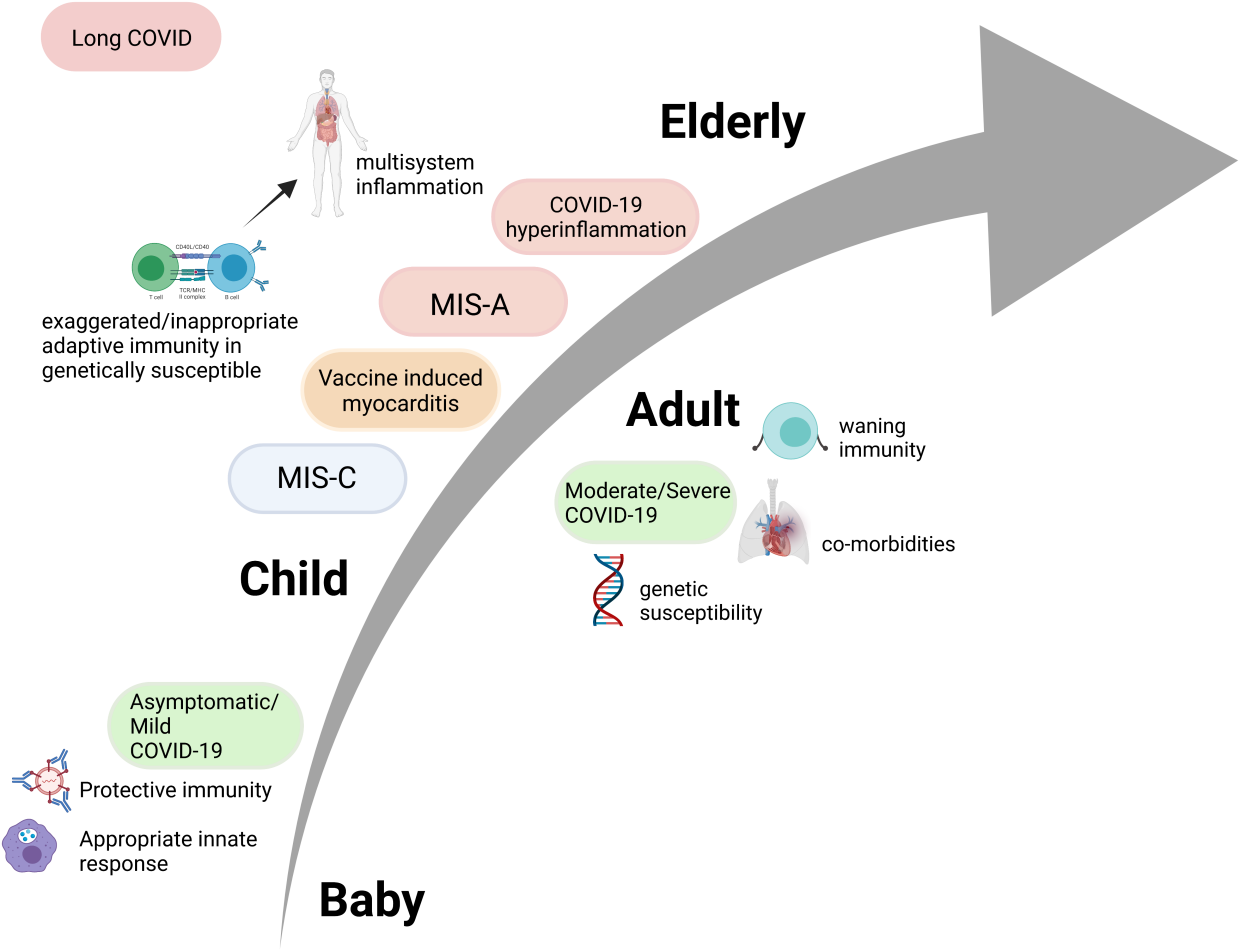
Factors influencing the spectrum of SARS‐CoV‐2‐associated disease in children and adults. MIS‐C, Multisystem Inflammatory Syndrome in Children; MIS‐A, Multisystem Inflammatory Syndrome in Adults.

There appear to be multiple phases of the immune response to SARS‐CoV‐2. In adults, the first phase is a protective immune response aimed at eliminating the virus.^24^ Some patients progress on to a second phase of illness characterised by an over‐activated or dysregulated host response and more severe multisystem disease.^24^ The third phase of illness has also been described, this is the postacute sequalae, termed ‘Long COVID’.^25^ While a universal case definition is yet to be determined, the syndrome is characterised by the persistence of generalised symptoms (e.g. fatigue, chest tightness, difficulty concentrating) weeks or months following acute SARS‐CoV‐2 infection,^25^ with recent data suggesting a greater prevalence in adults (estimated % of self‐reported symptoms in a 4‐week period in the UK: 2–16 years, ~0.4%; 17–34 years ~1.8%; 35–69 years ~2.3%^26^). In comparison, most children appear to mount an effective initial response to SARS‐CoV‐2 infection, as evidenced by low hospital admission rates of paediatric COVID‐19, but a small proportion can develop a later hyperinflammatory response resulting in MIS‐C^6^ (Figure 2).

Both MIS‐C and severe COVID‐19 patients have persistent fevers, systemic inflammation, hypotension and deranged coagulation, which can progress to multiorgan failure and death. These similarities suggest comparable underlying mechanisms, whereby a dysregulated host immune response and over‐production of inflammatory cytokines results in a cytokine storm leading to systemic inflammation and tissue damage.^27^

### Pre‐existing immunity

One of the factors influencing the heterogeneous outcomes following SARS‐CoV‐2 infection may be the effects of pre‐existing immunity from previous exposure to seasonal coronaviruses (e.g. HCoV‐229E, HCoV‐HKU1, HCoV‐NL63 and HCoV‐OC43). Seasonal coronaviruses circulate globally, causing self‐limiting upper respiratory tract illnesses in most individuals.^28^ Whilst the majority of primary infections occur in childhood, high rates of re‐infection have been reported in adults. Adults living with children have increased exposure to viruses and hence enhanced cross‐reactive immunity, leading to progressively reduced risk of SARS‐CoV‐2 infection with the increasing number of children in the household (adjusted Hazard Ratio per child 0.93; 95% CI 0.88–0.98).^29^

In addition, individuals with evidence of prior seasonal coronavirus infection (within the 3 years of SARS‐CoV‐2 infection) experienced less severe COVID‐19 compared to those without, including lower odds of requiring intensive care (OR 0.1, 95% CI 0.0–0.7) and better survival probability (HR 0.3, 95% CI 0.1–0.7).^30^ To understand the age‐related differences seen in COVID‐19 disease outcomes, Ng *et al*. compared the proportion of cross‐reactive anti‐S antibodies to SARS‐CoV‐2 in uninfected children and adults. 62% of children were found to have cross‐reactive antibodies compared with 5% of adults; these antibodies had neutralising effects on SARS‐CoV‐2 infection *in vitro*.^31^ However, this is inconsistent with findings from Lv *et al*., who report that while cross‐reactive antibodies are common, they are rarely neutralising.^32^ Consiglio *et al*. found an absence of IgG antibodies to HCoV‐HKU1 and beta coronaviruses among children with MIS‐C contrasted with high prevalence in healthy controls and those with KD and acute COVID‐19. This raises the possibility of prior exposure to seasonal coronaviruses protecting children from developing MIS‐C.^23^ In contrast, a smaller study by Sermet‐Gaudelus *et al*. demonstrated that prior exposure to seasonal coronaviruses did not influence the risk of MIS‐C or affect the humoral response to SARS‐CoV‐2.^33^

### Possible mechanisms of protective immunity

Potential protective effects may not be limited to antibody alone. Although the majority of SARS‐CoV‐2 anti‐spike antibodies are produced by naïve B cells in adults, differences in antigen‐specific memory B cells in the blood and lymphoid tissues from prepandemic children and adults,^34^ including higher frequencies of class‐switched convergent B‐cell clones against SARS‐CoV‐2 variants, may indicate that children clear a novel infectious agent more efficiently than adults due to imprinting of antibody responses.

Memory T cells play a protective role in a variety of infections, such as influenza. Children and adults both develop protective T cell responses following infection with SARS‐CoV‐2. SARS‐CoV‐2 cross‐reactive T cells (CD4^+^ > CD8^+^ ^35^) have been reported in 20–50% of unexposed adults.^36^, ^37^ These memory T cells recognise epitopes of both SARS‐CoV‐2 and seasonal coronaviruses, potentially providing cross‐protection.^38^ While such studies have not yet been performed in children, this protective mechanism could similarly contribute to mild illness seen in the paediatric population.

Innate immune imprinting, or ‘trained immunity’, where a primary response to a pathogen results in cross‐protection against other pathogens, is well described, particularly in relation to the Bacille Calmette‐Guerin (BCG) vaccine.^39^ Epidemiological evidence suggests that the BCG vaccine may protect against COVID‐19^40^; however, these studies may not account for all relevant confounders. Although clinical trials are underway to test this hypothesis, BCG vaccination is not currently recommended for the prevention of COVID‐19.

### Understanding the pathogenesis of MIS‐C

Similarities with other conditions, including macrophage activation syndrome, toxic shock syndrome and sepsis, have led research groups to explore various ‘immune readouts’ and immune responses associated with these conditions, both to understand the pathogenesis of MIS‐C, but also to guide diagnosis and treatment. These include cytokine measurements and determining whether innate or adaptive immune responses play a greater role in pathogenesis. Here we summarise the literature to date.

### Cytokine response

A number of small studies have compared cytokine profiles in MIS‐C and COVID‐19 with heterogenous findings. Overall, there is a marked inflammatory profile with a reflected anti‐inflammatory response (including IFN‐γ, IL‐1B, IL‐6, IL‐8, IL‐17, TNF‐α, IL‐10) in all cohorts (adult COVID‐19,^23^, ^41^, ^42^, ^43^ severe paediatric COVID‐19^43^, ^44^ and MIS‐C),^22^, ^23^, ^44^ with some possible differences between MIS‐C and COVID‐pneumonia.^44^ IFNγ has been found at similar or higher levels in MIS‐C than in adult COVID‐19 cases,^43^ and IL‐17, IL‐10 and TNF‐α appear to be higher in MIS‐C than either adult or paediatric severe COVID‐19.^43^, ^44^ In one cohort, proinflammatory cytokines (TNF‐α, IL‐6, IL‐10 and CXCL10) were significantly higher in children with moderate to severe disease in comparison to children with mild disease, particularly those with cardiac involvement.^45^ Chemokines involved in the recruitment and activation of a variety of immune cells (T cells, NK cells, neutrophils and monocytes) are also increased in MIS‐C.^46^

Given the predominance of GI symptoms, it is noteworthy that cytokines that enhance mucosal immunity (IL‐17A, CCL20 and CCL28) were raised in MIS‐C.^46^ Yonker *et al*. hypothesise a role for GI involvement and ongoing mucosal exposure to SARS‐CoV‐2, reporting persistently high serum anti‐SARS‐CoV‐2 IgA and IgM levels in MIS‐C. Furthermore, they propose that the hyperinflammatory response is triggered by the entry of viral antigens through mucosal breaches as demonstrated by detectable levels of SARS‐CoV‐2 RNA in stool samples in the majority of MIS‐C patients and higher levels of zonulin (a marker of increased intestinal permeability) in MIS‐C than in controls (*P* = 0.003).^47^

### Innate responses

As children predominantly experience mild or asymptomatic upper respiratory symptoms following infection with SARS‐CoV‐2, initial hypotheses suggested that an early and robust innate response is key in determining COVID‐19 disease severity. This naturally led to the plausibility of the importance of an excessive innate immune response in the development of MIS‐C.

The release of type 1 Interferons (IFNs) is an important part of innate immunity, as along with other cytokines (TNF‐α, IL‐1, IL‐6 and IL‐18), multiple antiviral actions result in a suppressed viral load and activation of the adaptive immune response.^48^ Cases of severe and fatal COVID‐19 have been shown to have reduced type 1 and 3 IFN responses.^49^, ^50^, ^51^ The significance of an adequate IFN response is highlighted further by Zhang *et al*. who identified inborn mutations in type I IFN signalling in 3.5% (23/659) of lethal cases,^52^ and the work by Bastard *et al*. demonstrating autoantibodies against type 1 IFN in 13% (135/987) of patients with severe COVID‐19.^53^ It has previously been shown that old age,^54^ obesity^55^ and atherosclerosis^56^ are associated with an impaired IFN response, while pubertal development and female sex are associated with an increased type 1 interferon response,^57^ which could contribute to age‐related differences in severity of COVID‐19 disease.

Carter *et al*. performed longitudinal immunophenotyping of innate immune cells in MIS‐C patients (acute *n* = 23, resolution *n* = 14, convalescence *n* = 10) and healthy paediatric controls (*n* = 7).^22^ They showed increased neutrophil and monocyte activation in the acute phase of MIS‐C compared with healthy controls, in keeping with observational data. Moreover, the surrogate markers of antigen presentation (HLA‐DR and CD86) in monocytes and dendritic cells were reduced, which could suggest either delayed or improper priming of adaptive immunity in children with MIS‐C or reflect regulatory immune responses in the face of uncontrolled inflammation.^22^ Ramaswamy *et al*. support these findings, with gene expression and protein studies showing reduced HLA‐DR and CD86 in monocytes of MIS‐C patients (*n* = 10) compared with healthy paediatric controls (*n* = 6).^58^ Moreover, the activation of these monocytes was dysregulated and revealed an elevated sepsis signature, similar to what is seen in COVID‐19 patients. Postinflammatory changes were apparent in myeloid cells, including upregulated S100A alarmin transcript and protein levels in neutrophils and monocytes.^58^ Finally, MIS‐C patients had high frequencies of NK cells with increased expression of cytotoxicity genes, compared with controls.^58^

Gruber *et al*. compared differences in innate immune cells in young adults with COVID‐19 (*n* = 7), MIS‐C (*n* = 9) and healthy paediatric controls (*n* = 5) using mass cytometry.^46^ They showed comparable levels of plasmacytoid dendritic cells and nonclassical monocytes (CD16^+^) in both patient groups, which were significantly lower than that in healthy controls. However, levels of CD56^lo^ cytotoxic NK cells were much lower in MIS‐C than in both healthy controls and COVID‐19 adults.^46^ Further phenotyping of immune cells in MIS‐C suggested that activation and chemotaxis of neutrophils and monocytes, and extravasation of T and NK lymphocytes, play a role in disease pathology.^46^

It would appear that there is a ‘goldilocks’ effect for the innate immune response to SARS‐CoV‐2, with a prolonged delay allowing uninhibited SARS‐CoV‐2 replication followed by a compensatory enhanced response and subsequent exaggerated adaptive immune response, which may contribute to the immunopathology seen in COVID‐19 hyperinflammation.

An impaired innate immune response can hypothetically result in two outcomes: asymptomatic/mild disease or severe COVID‐19. If the innate immune response is only mildly suppressed and the subsequent adaptive immune response is timely and appropriate, the infection can be controlled, resulting in mild disease. However, a prolonged delay in innate immunity not only allows uninhibited SARS‐CoV‐2 replication but results in a compensatory prolonged and enhanced innate immune response. This is then associated with a delayed and/or exaggerated adaptive immune response, which may contribute to the immunopathology seen in COVID‐19 hyperinflammation.^36^, ^59^ Although MIS‐C occurs 3–6 weeks after initial infection, the role of the innate immune response in driving immunopathology is of clear interest.

### B‐cell/antibody response

The role of ‘super‐antigens’, autoantibodies and B cells in the pathogenesis of MIS‐C and the hyperinflammatory phase of COVID‐19 in adults has been of great interest to many groups. Studies predominantly conducted in adults have led to the conclusion that the antibody response is not the major determinant of virological control or outcomes in COVID‐19 infection.^36^

Weisberg *et al*. showed similar anti‐spike protein responses in children with MIS‐C, acute COVID‐19 and adult convalescent plasma donors; however, adults with severe COVID‐pneumonia had the highest levels.^60^ Anti‐nucleocapsid antibodies were higher in adults than children, with the potential explanation that exposure to this antigen is driven by the lysis of infected cells. Although children produce neutralising antibodies following COVID‐19 of varying severity,^61^ adults overall had higher levels of neutralising antibodies than children, and no difference was noted between children with MIS‐C and acute COVID‐19.^60^

B‐cell lymphopenia has been noted in MIS‐C,^22^ although Gruber *et al*. reported broadly normal B‐cell subpopulation frequencies in MIS‐C.^46^ Elevated plasmablast frequencies have been identified both in children with acute COVID‐19^62^ and MIS‐C,^22^, ^58^, ^62^ in particular IgG1 and IgG3 plasmablasts.^58^ Given the likely weeks‐long period between SARS‐CoV‐2 exposure and development of MIS‐C, the elevation in plasmablasts supports a persistent B‐cell response. Interestingly, plasmablast frequencies declined with clinical improvement in MIS‐C, while the changes in plasmablast frequencies in paediatric COVID‐19 were variable.^62^ No correlation was noted between plasmablast frequencies and titres of specific antibody.^62^ Similar to adults with acute COVID‐19, children with MIS‐C have low proportions of plasmablasts displaying somatic hypermutation, although this is higher in severe than nonsevere MIS‐C.^58^

Three studies have investigated autoantibody presence and spectrum in children with MIS‐C, although it is important to note that the presence of autoantibodies does not confirm a pathological role. Gruber *et al*. found nearly 200 IgG peptide autoantigen candidates and over a 100 IgA candidates.^46^ These included anti‐La and anti‐Jo‐1, associated with systemic lupus erythematosus (SLE). They reported enrichment in peptides derived from endothelial, cardiac and gastrointestinal tissues and immune cell mediators (including IFNγR2).^46^ Around 15% of candidates could be validated through an approach that detects only linear epitopes.^46^ Consiglio *et al*. noted enrichment for autoantigens involved in lymphocyte activation processes, signalling phosphorylation and cardiovascular development.^23^ Ramaswamy *et al*. found that IgG from severe (*n* = 3) but not moderate (*n* = 3) MIS‐C patients bound cardiac microvascular endothelial cells.^58^

As with KD, the hypothesis of a superantigen‐driven disease has been raised for MIS‐C, and for the hyperinflammatory delayed illness of adult COVID‐19. Porritt *et al*. undertook a study of T‐cell receptor repertoire in 20 patients with MIS‐C and 14 febrile controls.^63^ T‐cell receptor B V locus usage clustered distinctly in febrile patients compared with MIS‐C.^63^ Three specific loci were found to be over‐represented in MIS‐C, including TRBV11‐2, confirmed by Ramaswamy *et al.,* ^58^ and TRBV24‐1, also described in adult severe COVID‐19. TRBV11‐2 usage correlated with TNF‐α, IFNγ, IL‐6 and IL‐10 concentrations.^63^ In support of a superantigen hypothesis (which typically selects for specific V loci, but not for J loci and CDR3 sequences), CDRs were distinct between patients with TRBV11‐2 and CDR lengths evenly distributed with J gene usage showing no bias.^63^ No associations with HLA class II alleles could be found, as might be expected, but HLA class I alleles A02, B35 and C04 were present in all four severe patients with TRBV11‐2 expansion. Yonker *et al*. provide further support for this theory, by demonstrating a potential mechanism for the persistence of SARS‐CoV‐2 antigen through increased gut permeability and a strong correlation between S1 antigenemia and expression of TRBV11‐2 in MIS‐C (Pearson's correlation, *r* = 0.89, *P =* 0.0005).^47^ Taken together with supportive structural evidence for a super‐antigenic region in S1,^63^ this clearly demands further investigation and identifies a potential similarity underlying the hyperinflammation of adult COVID‐19 and MIS‐C.

### T cell responses

Marked lymphopaenia in both severe COVID‐19 and MIS‐C has led to an interest in T cell immunology in the pathogenesis of these conditions.

Vella *et al*. found that children both with acute COVID‐19 (*n* = 14) and MIS‐C (*n* = 9) had lymphopenia, although more pronounced in MIS‐C, and that all had high levels of CD4^+^ T cell proliferation, with both CD4^+^ and CD8^+^ T cell proliferation in MIS‐C exceeding that in adult COVID‐19.^62^

Most studies have found similar T cell subset distributions using both single‐cell transcriptomic and cell surface‐marker approaches. Significant findings in comparison with healthy controls are consistent with T‐cell activation. These include reduced γδ T cells^22^, ^46^ and naive T cells,^58^ expanded CD4^+^CCR7^+^ T cells^22^ and tissue‐homing phenotypes of activated T cells.^58^

Consiglio *et al*. showed that children with both acute COVID‐19 (*n* = 41) and MIS‐C (*n* = 13) had lower proportions of T follicular helper cells than healthy controls (*n* = 19) and children with KD (*n* = 28).^23^ Cotugno *et al*. showed this only for children both infected with SARS‐CoV‐2 and negative for neutralising antibodies (29/66 positive for SARS‐CoV‐2).^61^ Vella *et al*. did not find a difference, although their healthy controls were adults.^62^ The abundance of SARS‐CoV‐2 antigen‐specific T cells correlated with titres of neutralising antibodies,^61^ and the frequency of activated CD4^+^ T cells with plasmablasts.^58^ Patients positive for neutralising antibodies had lower circulating terminally differentiated memory T cells (T_EMRA_).^61^

To summarise, there appears to be a progressive and appropriate adaptive immune response measurable in both COVID‐19 and MIS‐C, described by falling naive T cells, activated and proliferating CD4^+^ T cells, and correlation with the development of neutralising antibodies. A tissue‐homing phenotype on the activated CD4^+^ T cells, alongside consistently described lymphopaenia in blood samples may suggest that this adaptive immune response is focused on tissues such as GI, heart and lungs, contributing to the pathology of disease.

### Response to vaccines

To date, multiple vaccines have been approved for use in SARS‐CoV‐2^64^ (e.g. Moderna,^65^ Pfizer,^66^ AstraZeneca,^67^ Sputnik^68^). Presently, approved mRNA vaccines confer superior efficacy versus adenovirus vaccines in adults (90–95% vs 71–91%). Early safety and efficacy data in children are reassuring; longer term safety is awaited.^65^, ^66^, ^67^, ^68^ Interestingly, early trial results suggest that 12‐ to 15‐year‐olds who received two doses of the Pfizer‐BioNTech vaccine mounted superior antibody levels than 16‐ to 25‐year‐olds in earlier trials, which raises the possibility that lower doses may confer similar immunity.^69^ Given the much lower morbidity and mortality from SARS‐CoV‐2 infection in children, decisions regarding vaccination are finely balanced, and whether to account for secondary impacts like mandated school absence, and onward spread is hotly debated. An important question is whether vaccination may protect from, or promote MIS‐C, and this will be carefully monitored. Early reports have raised concerns about vaccine‐induced myocarditis in children and young adults; however, this complication is extremely rare and in the vast majority has been easily treated.^70^ It is tempting to conclude this may relate in some ways to MIS‐C; however; this is a single‐system illness without hyperinflammation and may be quite distinct. There are also growing concerns around the very rare observation of blood clots linked to the Johnson and Johnson and Oxford‐AstraZeneca vaccines, and at present, these trials in the paediatric population have been paused. Further data on vaccine safety and efficacy will help countries to assess the balance of benefits and harms of paediatric vaccination and whether to make blanket recommendations.

### Treatment of MIS‐C

In the absence of data from randomised trials, and faced with a serious new disease, it was not surprising that paediatricians worldwide have adopted a range of anti‐inflammatory and immunomodulating agents for treatment of MIS‐C. The international Best Available Treatment Study (BATS), which used propensity weighting to account for differences in severity, has provided evidence that the most commonly used immunomodulator treatments [intravenous immunoglobulin (IVIG), corticosteroids or combination of IVIG and corticosteroids] are associated with more rapid resolution of inflammation than no immunomodulator treatment.^71^ Other cohort studies from France^72^ and the USA^73^ have demonstrated more rapid resolution of cardiac dysfunction in patients initially treated with corticosteroids and IVIG as opposed to IVIG alone. While the BATS study did not find differences between these three treatments for the rate of progression to, or recovery from, organ failure, evidence of benefit from steroids compared with IVIG was found in the subgroup of children meeting the WHO MIS‐C criteria.^71^

As IVIG is expensive, and less available in many countries, there is great interest in whether steroids might be used as a cheaper and more readily available alternative to IVIG. There is currently insufficient data to establish that steroids alone are equivalent to IVIG, or to recommend their use as an alternative to IVIG‐containing regimes. Answers to this question are likely to come from both randomised studies now underway (RECOVERY) or from further analysis or pooling of ongoing cohort studies.

### Clinical implications, ongoing studies, open questions

Since its recognition, a growing body of research has emerged to allow clinical and immunological characterisation of MIS‐C, providing some diagnostic and therapeutic guidance for clinicians. However, the significance of many of these findings along with the clinical implications is yet to be determined. The majority of work on COVID‐19 immunology focuses on adults or children alone, and studies considering both are limited and have small sample sizes. Further work with careful design and greater numbers of patients is needed to address some key questions, which remain unanswered (Box 1).

Many large epidemiological, observational and clinical trials (e.g. BATS,^74^ RECOVERY,^75^ DIAMONDS,^76^ BPSU,^77^ ISARIC^78^) continue and are hoped to provide greater insight. Preliminary data generated by such studies have demonstrated the value of multidisciplinary and international collaborations for generating high‐quality research at an accelerated pace.

### Concluding paragraph

Following SARS‐CoV‐2 infection, children and adults initially present with either no or mild symptoms (e.g. fever, cough, rhinorrhoea). However, unlike adults, only a small minority of children progress to severe disease requiring hospitalisation. While acute respiratory COVID‐19 is clinically and immunologically distinct from the postinfectious inflammatory syndrome, similarities have been drawn between severe COVID‐19, MIS‐C and other inflammatory conditions such as KD, with the suggestion of comparable underlying immunopathology. Therefore, comparison of the immunology in children and adults with varying severity of COVID‐19, and children with MIS‐C and other inflammatory conditions, will provide insight into disease pathophysiology. Currently available data from vaccine studies are encouraging with reports of effective and long‐lasting immunity. However, early reports of myocarditis in children and young adults following mRNA vaccines suggest that introduction of COVID‐19 vaccines into the child population will need to be undertaken cautiously, with close monitoring of adverse effects. Recognition of myocarditis following vaccines based on SARS‐CoV‐2 spike protein sequences may provide a clue to the mechanisms of cardiac injury in MIS‐C and acute COVID‐19 and requires detailed investigation. Finally, as it is less than 2 years since COVID‐19 was recognised, and under 18 months since the recognition of MIS‐C, it is remarkable how much has been learned about the immunology and spectrum of diseases caused by SARS‐CoV‐2. Immunological and genetic studies containing larger numbers of well‐characterised children across the spectrum of asymptomatic SARS‐CoV‐2 infection to severe COVID‐19 or MIS‐C are likely to define the mechanisms underlying the different host responses to this new pandemic virus.

## Conflict of interest

The authors declare no conflicts of interest.

## AUTHOR CONTRIBUTIONS

**Harsita Patel:** Writing – original draft; writing – review and editing. **Andrew McArdle:** Writing – original draft; writing – review and editing. **Eleanor Seaby:** Writing – original draft; writing – review and editing. **Michael Levin:** Writing – review and editing.
